# Measurement of coronary calcium scores or exercise testing as initial screening tool in asymptomatic subjects with ST-T changes on the resting ECG: an evaluation study

**DOI:** 10.1186/1471-2261-7-19

**Published:** 2007-07-13

**Authors:** Christiane A Geluk, Riksta Dikkers, Jan A Kors, René A Tio, Riemer HJA Slart, Rozemarijn Vliegenthart, Hans L Hillege, Tineke P Willems, Paul E de Jong, Wiek H van Gilst, Matthijs Oudkerk, Felix Zijlstra

**Affiliations:** 1 Department of Cardiology, Thoraxcenter, University Medical Center Groningen, University of Groningen, The Netherlands; 2 Department of Radiology, University Medical Center Groningen, University of Groningen, The Netherlands; 3 Department of Medical Informatics, Erasmus MC-University Medical Center Rotterdam, Rotterdam, The Netherlands; 4 Department of Nuclear Medicine and Molecular Imaging, University Medical Center Groningen, University of Groningen, The Netherlands; 5 Departement of Internal Medicine, Division of Nephrology, University Medical Center Groningen, University of Groningen, The Netherlands; 6 Department of Clinical Pharmacology, University Medical Center Groningen, University of Groningen, The Netherlands

## Abstract

**Background:**

Asymptomatic subjects at intermediate coronary risk may need diagnostic testing for risk stratification. Both measurement of coronary calcium scores and exercise testing are well established tests for this purpose. However, it is not clear which test should be preferred as initial diagnostic test. We evaluated the prevalence of documented coronary artery disease (CAD) according to calcium scores and exercise test results.

**Methods:**

Asymptomatic subjects with ST-T changes on a rest ECG were selected from the population based PREVEND cohort study and underwent measurement of calcium scores by electron beam tomography and exercise testing. With calcium scores ≥10 or a positive exercise test, myocardial perfusion imaging (MPS) or coronary angiography (CAG) was recommended. The primary endpoint was documented obstructive CAD (≥50% stenosis).

**Results:**

Of 153 subjects included, 149 subjects completed the study protocol. Calcium scores ≥400, 100–399, 10–99 and <10 were found in 16, 29, 18 and 86 subjects and the primary endpoint was present in 11 (69%), 12 (41%), 0 (0%) and 1 (1%) subjects, respectively. A positive, nondiagnostic and negative exercise test was present in 33, 27 and 89 subjects and the primary endpoint was present in 13 (39%), 5 (19%) and 6 (7%) subjects, respectively. Receiver operator characteristics analysis showed that the area under the curve, as measure of diagnostic yield, of 0.91 (95% CI 0.84–0.97) for calcium scores was superior to 0.74 (95% CI 0.64–0.83) for exercise testing (p = 0.004).

**Conclusion:**

Measurement of coronary calcium scores is an appropriate initial non-invasive test in asymptomatic subjects at increased coronary risk.

## Background

In selected asymptomatic subjects with an intermediate coronary risk profile, non-invasive testing may be required for coronary risk stratification [1-5]. Guidelines recommend the use of exercise testing [2,6,7] and measurement of coronary calcium scores [8], since the predictive values of both non-invasive tests for future coronary events have been well established in asymptomatic subjects at intermediate risk [7,9-11]. However, guidelines do not recommend one of these tests as initial screening tool in these subjects. Although an abnormal exercise test result is widely accepted as indication for coronary angiography (CAG)[12], the indications for an invasive diagnostic or therapeutic procedure have not yet been defined for subjects with high calcium scores. This may be of particular importance for subjects with calcium scores ≥400, since these subjects are at a high annual risk of cardiac events of 4.8% [9]. So far, no head to head studies have compared exercise testing with measurement of calcium scores as initial tool in the evaluation of coronary artery disease (CAD).

Our aim was to investigate the diagnostic yield of coronary calcium scores and exercise testing in asymptomatic subjects with an intermediate coronary risk profile. Therefore, in a population based cohort study, all 12-lead rest ECGs with ST depression (defined as Minnesota codes 4.1-2 (>0.5 mm ST-junctional depression)), T-wave inversion (codes 5.1-2 (T wave inversion ≥1.0 mm)) or with an abnormal frontal T-axis (-180° to -15° and 105° to 180°) [13-17] were selected, after exclusion of the ECGs with non-interpretable ST segments during the exercise test by a clinical cardiologist [7]. These subjects are representative for a study population at intermediate coronary risk, due to the selection from a low risk population and in the presence of ≥1 high risk characteristic [13-18]. First, we investigated the prevalence of documented CAD. Second, we investigated the invasive therapeutic implications according to coronary calcium scores and exercise test results.

## Methods

### Subjects

Asymptomatic male and female subjects with ST-T changes on a 12-lead resting ECG were selected from the prospective population based Prevention of REnal and Vascular ENdstage Disease (PREVEND) cohort study in Groningen, the Netherlands. The primary aim of this cohort study is to assess the value of urinary albumin excretion in relation to cardiovascular and renal risk. In addition to the ECG, collected data include medical history, demographics, biometric data, urine- and blood collections and laboratory measurements. The first visit has taken place between 1997–1998. Subjects for the current study were selected after the second visit (2001–2003). Exclusion criteria were previous manifestations of coronary heart disease (myocardial infarction, revascularization procedure, or Q waves on the ECG) or coronary angiography (CAG); age >70 years; and subjects in whom the ST-T segment was not interpretable during the exercise test due to atrial fibrillation or left bundle branch block (LBBB) or ST depression >1 mm at 80 msec after the J point [7]. For details on the PREVEND study design we refer to earlier publications [19]. All participants underwent measurement of calcium scores by EBT and exercise testing. This PREVEND substudy was approved by the medical ethics committee and conducted in accordance with the guidelines of the declaration of Helsinki. All participants have given written informed consent.

### Electrocardiography

Standard 12-lead ECGs were recorded with Cardio Perfect equipment (Cardio Control, Delft, The Netherlands), stored digitally, and classified according to the Minnesota code, using the computer program MEANS (Modular ECG Analysis System) [20]. Signal analysis and classification of MEANS have been extensively evaluated [21,22]. ST-T segment changes were defined by Minnesota codes 4.1-2 (ST-junctional depression = 0.5 mm) and 5.1-2 (negative T-wave ≥1 mm) or abnormal mean frontal T-axis (-180° to -15° and 105° to 180°) [13-18]. T axes were computed from vectorcardiographic X, Y and Z leads, which can, in good approximation, be reconstructed from the standard ECG leads [23]. The mean spatial axis was obtained by vectorially adding the instantaneous heart vectors during the T wave. The mean frontal T axis is the angle between the X axis and the projection of the mean spatial T axis on the frontal XY plane. Q waves were defined by Minnesota codes 1.1–1.3 [17]. All ECGs were reviewed by a senior clinical cardiologist in order to exclude the ECGs of which the ST-T segment was not interpretable during the exercise test.

### Measurement of coronary calcium scores

Coronary calcium was measured using electron beam tomography (EBT) (e-Speed, GE Medical Systems, South San Francisco, USA). According to subjects' weight and size the beam speed was set to 50 ms (for small or slender patients) or 100 ms (for larger patients). Prospective ECG triggering was used and set at 42% of the R-R interval. Scans were made without the use of a contrast agent with 130 kV and 895 mAs. A single collimation of 3.0 mm and an increment of 3.0 mm was applied. Total radiation exposure was <1 mSv for each patient. The coronary calcium score was obtained by multiplying each area of interest with a factor indicating peak density within the individual area, as was proposed by Agatston [24].

### Exercise testing

As is common practice in the Netherlands, all exercise tests were performed on a bicycle. Exercise tests were performed in accordance with the guidelines for exercise testing [6,7]. All exercise tests were independently reviewed by a cardiologist (RT) and a research physician (CG), who reached consensus in all cases. Exercise test end points were defined as follows: positive, in case of ECG evidence of myocardial ischemia (≥1.0 mm horizontal shift of the ST segment at 80 msec after the J point compared to the baseline ECG) and/or in case of 30 mmHg decrease in systolic blood pressure and/or ventricular arrhythmia and/or typical angina; intermediate, in case of <1.0 mm ST depression as compared to baseline and/or aspecific anginal complaints in the absence of ECG evidence of ischemia; negative, in the absence of any of the above mentioned criteria; and non-interpretable, if <85% of the age- and sex- predicted heart rate or a rate pressure product <18,000 was achieved. Intermediate and non-interpretable results are considered as "nondiagnostic test results".

### Protocol

**Figure 1:**
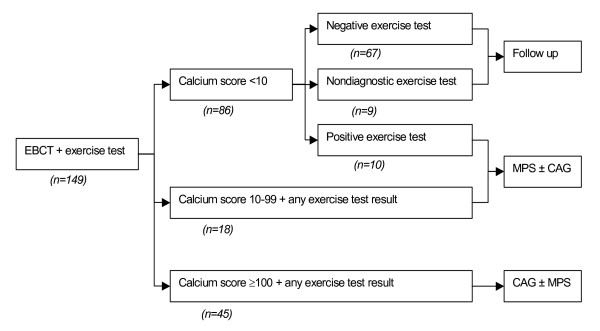
Decision protocol*. *see text for explanation. Abbreviations: CAG, coronary angiography; MPS, myocardial perfusion scintigraphy.

All patients underwent measurement of coronary calcium scores by EBT and exercise testing. Test performance was evaluated according to the decision protocol as given in figure 1. In case of calcium scores ≥10, or positive exercise test result, CAG or MPS was recommended to evaluate the presence of obstructive CAD (figure 1). In subjects with calcium scores <10 and a negative or nondiagnostic exercise test result, a test to document obstructive CAD was not recommended. In this population the presence of obstructive CAD is almost fully excluded due to the high negative predictive value of low calcium scores on CAD [9,10,25]. In subjects with calcium scores <10 and a positive exercise test and in subjects with calcium scores 10–99, the first choice recommended test was myocardial perfusion scintigraphy (MPS), followed by CAG in case of abnormal results. In subjects with calcium scores 100–399 and in subjects with calcium scores ≥400, the first choice recommended test was CAG.

### Endpoints

The primary endpoint was defined as documented CAD, i.e. presence of obstructive significant CAD (≥50% luminal obstruction), based on CAG, or MPS, in case CAG was not available. Myocardial perfusion scintigraphy was performed as previously described [26]. All CAGs were re-analysed by a senior cardiologist (FZ), without knowledge of the clinical data. By qualitative analysis the coronary arteries were graded as follows: normal coronary arteries, defined as the absence of any coronary lesion; non-obstructive CAD, if maximal luminal obstructions were <50%; and obstructive CAD, if lesions obstructed the lumen ≥50% (i.e. documented CAD). The secondary endpoint was a class I or IIa indication for a revascularization procedure (percutaneous coronary intervention (PCI) or coronary artery bypass graft surgery (CABG)) according to the ESC and ACC/AHA guidelines for PCI and CABG [27-29]. Decisions to perform a revascularization procedure were taken by the Thoraxcenter multidisciplinary heart team. In case of obstructive CAD (= 50% luminal stenosis), either MPS, or fractional flow reserve (FFR) measurement [30,31] was performed to guide the decision for a revascularization procedure. An FFR <0.75 was an indication for a revascularization procedure. All subjects were followed for the occurrence of cardiac events, i.e. myocardial infarction or coronary death. Cardiac events were collected by review of the subject's medical record, questionnaire or telephone interview.

### Statistical analysis

Continuous data are expressed as mean ± standard deviation. Hypertension was defined as a systolic blood pressure >140 mmHg and/or diastolic >90 mmHg or use of antihypertensive medication. Left ventricular hypertrophy (LVH) on the ECG was defined according to the Cornell voltage-duration product [19]. Framingham risk estimations were calculated according to Wilson et al [32]. Since HDL cholesterol was not measured during the second visit, HDL cholesterol data of the first visit were used for the Framingham risk estimations. Significance was reached when p < 0.05. To compare the diagnostic yield of calcium scores with exercise test results, receiver operating characteristic (ROC) curve analysis was performed. We compared the area under the curves of both tests for the primary and the secondary endpoints. For the ROC analysis, subjects with calcium scores <10 and a negative or nondiagnostic exercise test and in whom MPS or CAG was not performed, were assumed to have no endpoints. Calculations were performed using the statistical package SPSS version 12.0 (SPSS, Chicago, USA) and STATA 9.0 (College Station, Texas, USA).

## Results

### Baseline characteristics

In 6,804 (99%) of 6,894 subjects participating in PREVEND between 2001–2003, a 12-lead resting ECGs was recorded. Of 481 (7%) subjects with ST-T changes on the ECG, 291 subjects had any of the following characteristics, namely previous manifestation of coronary heart disease; age >70 years; atrial fibrillation; LBBB or ST depression >1.0 mm at 80 msec after the J point. Of 190 subjects invited, 153 responded (81%) and were included. Baseline characteristics of the participants are shown in table 1. Of the 11 subjects with ECG criteria for LVH, 5 had calcium scores of 0 and 6 between 16 and 1313. The 10-year estimated Framingham risk was <10% in 49%, 10–20% in 22% and >20% in 29% of subjects.

**Table 1:** Baseline characteristics

**Characteristics**	**n = 153**
Age, mean (SD), y	56 (9)
Male gender, No. (%)	88 (58)
Blood pressure, mean (SD), mm Hg	
Systolic	133 (23)
Diastolic	76 (10)
Hypertension, No. (%)	75 (49)
Current smoking, No. (%)	30 (20)
Diabetes, No. (%)	15 (10)
History of Cerebrovascular accident, No. (%)	3 (2)
History of Peripheral Arterial Disease, No. (%)	2 (1)
History of Valvular Heart Disease, No. (%)	0 (0)
Total cholesterol, mean (SD), mmol/L	5.5 (1.1)
Albuminuria, median (interquartile range), mg/24 h	8.7 (5.9–17.3)
Medication, No. (%)	
Lipidlowering	29 (19)
Antihypertensive medication	62 (41)
Diuretics	37 (24)
Betablockers	24 (16)
ACE/AII blockers	30 (20)
Calciumantagonists	8 (5)
Aspirin	13 (9)
Antidiabetic treatment	15 (10)
Left ventricular hypertrophy criteria, No. (%)	11 (7)

Abbreviations: HDL, high density lipoprotein

SI conversion factor: to convert mmol/L to mg/dL, divide values for total cholesterol and HDL cholesterol by 0.0259 and divide values for triglycerides by 0.0113.

### Test results

Eighty-six (58%) participants had low calcium scores (<10), 18 (12%) had calcium scores 10–99, 29 (19%) participants had calcium scores 100–399 and 16 (11%) participants had high calcium scores (= 400). Exercise test characteristics are shown in table 2. Significant ST depression occurred in 19% of subjects and angina was present in 2%. Eighty-nine (60%) participants had a negative exercise test result, while 27 (18%) had a nondiagnostic and 33 (22%) had a positive exercise test result.

**Table 2:** Exercise test characteristics*

Rest	
Heart rate, per minute	75 (13)
Systolic blood pressure, mmHg	142 (23)
Diastolic blood pressure, mmHg	83 (11)
Exercise	

Heart rate, per minute	147 (27)
Systolic blood pressure, mmHg	205 (32)
Diastolic blood pressure, mmHg	90 (16)
Exercise capacity, Watt	151 (57)
Rate pressure product	30,141 (7,381)
Significant ST depression, No. (%)†	29 (19)
>30 mmHg decrease in blood pressure, No. (%)	3 (21)
Ventricular arrhythmia, No. (%)	5 (3)
Angina, No. (%)	3 (2)
Recovery	

Heart rate, per minute	97 (21)
Systolic blood pressure, mmHg	145 (37)
Diastolic blood pressure, mmHg	76 (19)

*Values are given in mean (SD) unless indicated.

†for definition please see methods section

### Endpoints

Four patients refused to undergo CAG or MPS as recommended by the decision protocol, namely one patient with calcium scores <10 and a positive exercise test; one patient with calcium scores 10–99 and a positive exercise test; one patient with calcium scores 10–99 and a negative exercise test; and one patient with calcium scores ≥400 and positive exercise test result. Therefore, outcome was obtained in 149 participants (100%) during 14 ± 3 months of follow up. No cardiac events occurred during follow up. The primary endpoint (documented CAD) was present in 24 (16%) participants. In 16 (11%) participants, the secondary endpoint, a Class I or IIa indication for revascularization procedure, was present according to the ESC and ACC/AHA guidelines for PCI and CABG in asymptomatic patients [27-29].

### The diagnostic yield of coronary calcium scores compared to the exercise test

**Figure 2:**
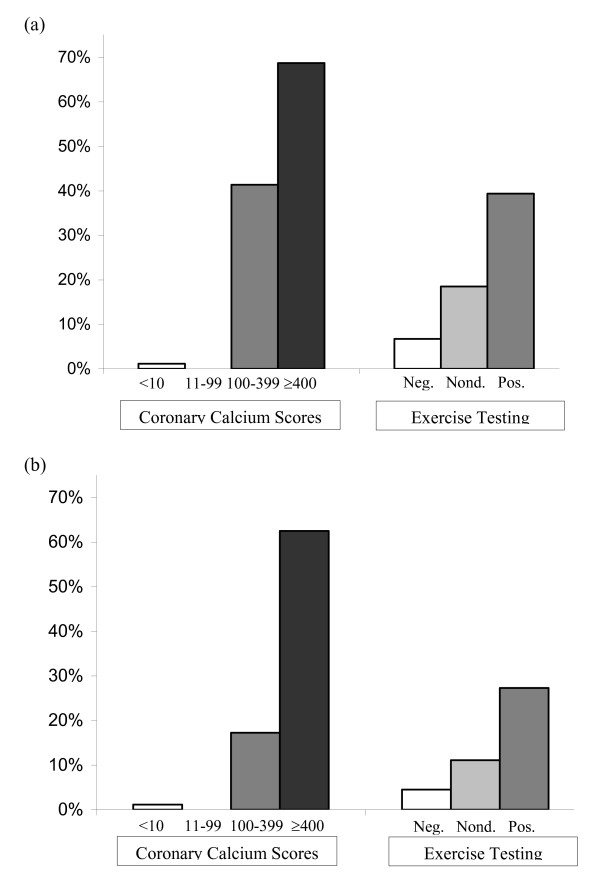
**Endpoints according to coronary calcium scores and exercise test results**. (a) primary endpoint (documented significant obstructive coronary artery disease) (b) secondary endpoint (Class I or IIa indication for revascularization procedure). Abbreviations: neg., negative; nond., nondiagnostic; pos., positive

**Figure 3:**
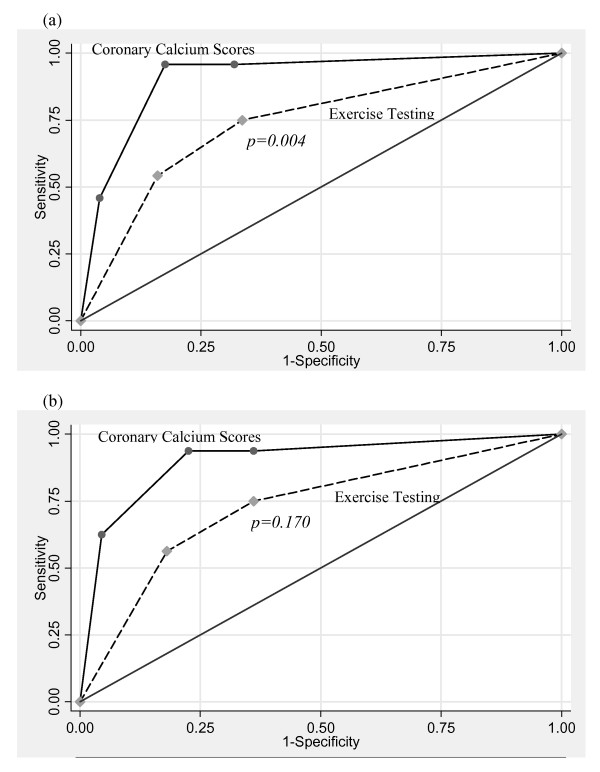
**Receiver operator characteristic curves**. (a) primary endpoint (documented significant obstructive coronary artery disease) Area under the curve for coronary calcium scores 0.91 (95% CI 0.84–0.97); Area under the curve for exercise test 0.74 (95% CI 0.64–0.83). (b) secondary endpoint (Class I or IIa indication for revascularization procedure) Area under the curve for coronary calcium scores 0.90 (95% CI 0.78–1.00); Area under the curve for exercise test 0.73 (95% CI 0.52–0.93)

Results are shown in table 3 and figure 2. The primary and secondary endpoints were present in, respectively, 69% and 63% of 16 subjects with calcium scores = 400 and in, respectively, 39% and 27% of 33 subjects with a positive exercise test. In 37% of the 33 subjects with a positive exercise test, the absence of CAD was confirmed by a low calcium score and/or normal coronary arteries at CAG. A false negative test result was observed in 1% of 86 subjects with calcium scores <10 and in 7% of 89 subjects with a negative exercise test result. The subject with calcium scores <10, a positive exercise test and obstructive CAD was a non-diabetic, male 56-year old subject, a past smoker, who underwent a revascularization procedure for a left main stenosis. With regard to the primary endpoint, ROC statistics show an increased diagnostic yield of calcium scores above exercise testing: the area under the curve is 0.91 (95% CI 0.84–0.97) for calcium scores versus 0.74 (95% CI 0.64–0.83) for exercise testing (p = 0.004; figure 3). With regard to the secondary endpoint, ROC statistics show a similar pattern, albeit not statistically significant: the area under the curve is 0.90 (95% CI 0.78–1.00) for calcium scores versus 0.73 (95% CI 0.52–0.93) for exercise testing (p = 0.170; figure 3).

**Table 3:** Endpoints

Calcium score		Exercise test result	No. (%)	No. of CAG*	No. of MPS*	No. with primary endpoint†	No. with secondary endpoint‡
<10	(n = 86)	Positive	10/86 (12%)	3	7	1	1
		Nondiagnostic	9/86 (10%)	-	-	0	0
		Negative	67/86 (8%)	-	-	0	0
10–99	(n = 18)	Positive	2/18 (11%)	1	1	0	0
		Nondiagnostic	8/18 (44%)	4	4	0	0
		Negative	8/18 (44%)	2	6	0	0
100–399	(n = 29)	Positive	14/29 (48%)	11	3	6	2
		Nondiagnostic	5/29 (17%)	3	2	2	1
		Negative	10/29 (34%)	7	3	4	2
≥400	(n = 16)	Positive	7/16 (44%)	7	0	6	6
		Nondiagnostic	5/16 (31%)	3	2	3	2
		Negative	4/16 (25%)	4	0	2	2

Abbreviations: CAG, coronary angiography; MPS, myocardial perfusion scintigraphy

*MPS was used for endpoint grading when CAG was not available

†primary endpoint, documented significant coronary artery disease

‡secondary endpoint, Class I or IIa indication for a revascularization procedure in asymptomatic patients

## Discussion

### Principal findings

Our results show that the diagnostic yield of measurement of coronary calcium scores for documented obstructive CAD is clearly superior to exercise testing in asymptomatic subjects at increased coronary risk. Furthermore, compared to exercise testing, high calcium scores identified a higher number of subjects with a class I or IIa indication for a revascularization procedure according to the ESC and ACC/AHA guidelines for PCI and CABG.

### General comments

Non-invasive testing, such as measurement of coronary calcium scores and exercise testing, have become well established tests for risk stratification in selected asymptomatic subjects encountered in clinical practice. [1,2,6-8] Both tests provide fundamentally different diagnostic information. Coronary calcifications are highly specific for atherosclerosis and a strong correlation with total plaque burden has been demonstrated [33,34]. Coronary calcifications parallel the development of atherosclerosis, with higher values present in men and in the elderly [35]. Higher amounts of coronary calcium have been associated with more severe CAD [33,34,36,37]. Coronary calcifications have been associated with hard as well as with soft plaques [38]. In ultrasound studies the sensitivity for the detection of soft plaques is lower than for hard plaques [38]. However, since the absence of coronary calcium has been associated with a negative predictive value of >95% for future coronary events [39,40], (soft) plaques maybe missed by EBCT have a limited clinical importance [5]. Absolute coronary calcium scores, as well as age- sex- specific percentiles, have been associated with the occurrence of future coronary events [35,39-41]. Measurement of coronary calcium scores therefore focuses on the detection of CAD, while the exercise test focuses on the detection of myocardial ischemia. The "anatomic approach" has the advantage that certainty on the absence of clinically important CAD is obtained when calcium scores equals zero. This is of clinical importance since many subjects at intermediate risk (a probability of a coronary event between 1–2% per year due to the presence of at least one high risk characteristic or based on Framingham scores [4,5]) do not have CAD [42-44].

In asymptomatic populations, guidelines traditionally focus on long-term risk assessment and prevention of future manifestations of coronary disease, while the role of invasive diagnostic and therapeutic procedures is hardly discussed [8,45]. Although high risk criteria on stress testing in asymptomatic subjects are accepted as indications for CAG [12], direct referral to CAG based on high calcium scores is generally believed to be inappropriate [12,46]. However, several arguments favor an invasive strategy in subjects with high calcium scores. A clear association has been demonstrated between calcium scores and the amount of myocardial ischemia [47-49] as well as the severity of CAD [33,34,36], which are the principle components of guideline recommendations for a revascularization procedure [27-29]. In addition, event free survival was decreased in asymptomatic subjects with high calcium scores and an abnormal myocardial perfusion test [49]. Clearly, all subjects with high calcium scores require aggressive secondary prevention, including treatment with cholesterol lowering, antihypertensive medications and aspirin. Our research protocol recommended performance of CAG in case of calcium scores = 400 or positive exercise test result. The decision to perform a revascularization procedure when a non-invasive stress test had not been performed prior to CAG, was guided by fractional flow reserve measurement during CAG [12,30,31]. Alternative diagnostic strategies may be the performance of CAG only after documentation of myocardial ischemia by non-invasive stress testing [46], or in combination with current generation CT-angiography. We agree that an invasive strategy is associated with a risk of complications and inappropriate revascularizations. The recent COURAGE trial has shown that some patients with stable CAD can be managed conservatively [50], and future guidelines may therefore be adapted. Our multidisciplinary study was based on the former ESC and ACC/AHA guidelines for PCI and CABG [27-29], and identified a substantial number of subjects with a class I or IIa indication for a revascularization procedure.

Twenty-nine subjects had a calcium score between 100–399. When compared to subjects with calcium scores = 400, less subjects had a primary endpoint (41% vs 68%) or an indication for a revascularization procedure (17% vs 63%). This finding is in line with previous findings on the increasing number of abnormal stress tests in case of higher calcium scores, namely in 18–60% of subjects with calcium scores = 400, compared to 7–23% of subjects with calcium cores of 100–399 [47-49]. Since the finding of myocardial ischemia in asymptomatic subjects with calcium scores >100 affects clinical outcome [49], non-invasive stress testing is warranted. This may be followed by an invasive strategy in case of abnormal test results, in addition to appropriate medical treatment. Further studies with larger numbers of patients are needed to evaluate these issues.

### Remarks and limitations

Thirthy-two patients (21.5%) used betablockers or calcium antagonists at the time of exercise testing. These medications may affect the maximal exercise heart rate [51]. This may have contributed to a non-interpretable result found in one case (0.7%). With regard to the exercise test, information on the Duke score and ST-T hysteresis were not measured. Unfortunately, individual FFR values were not registered. We used EBT to measure calcium scores. Due to recent improvements in ECG gating software, shorter scan times and higher resolutions, current generation multi detector CT also provides accurate calcium scores measurements with a radiation dose of 1.0 mSv [52]. Since multidetector CT scanners are more widely available than EBT, our results, when extended to multidetector CT scanners, may therefore influence clinical practice. The sensitivity of the exercise test was somewhat lower than expected from large symptomatic populations undergoing exercise testing and coronary angiography [53]. However, the test characteristics of our study were very comparable to the studies including only asymptomatic subjects [7,54-56]. The specificity for high calcium scores to detect significant CAD in our study population is similar to studies comparing calcium scoring and CAG in symptomatic patients [36,37]. This observation implies that the association between calcium scores and severity of CAD at CAG may be extendable to asymptomatic populations. The current study population was derived from the PREVEND population of subjects without previous documented coronary heart disease, which can be regarded as a low risk population since 3.3% experienced a first coronary event during 5.5 years of follow up [57]. The presence of ST-T changes on the resting ECG is a clear additional high risk characteristic, and therefore our population can be classified as intermediate risk [13-18]. The prevalence of coronary calcium scores, in particular with regard to the 30–50% of subjects having calcium scores <10, was comparable to other asymptomatic populations with at least one risk factor [42,43]. Our results are therefore applicable in asymptomatic populations, who are candidates for risk stratification, based on the presence of ≥1 high risk characteristic.

## Conclusion

Measurement of coronary calcium scores is an appropriate initial non-invasive test in asymptomatic subjects at increased coronary risk. Furthermore, invasive diagnostic and therapeutic procedures are indicated in a high number of subjects with coronary calcium scores ≥400.

## Abbreviations

CAD = coronary artery disease

CABG = coronary artery bypass graft surgery

CAG = coronary angiography

CT = computed tomography

EBT = electron beam tomography

MPS = myocardial perfusion imaging

PCI = percutaneous coronary intervention

PREVEND = Prevention of REnal and Vascular ENdstage Disease

ROC = receiver operating characteristics

## Competing interests

The author(s) declare that they have no competing interests.

## Authors' contributions

CAG, RD, JAK, RAT, RHJAS, HLH, RV, TPW, PEJ, WHG, MO, FZ were involved in the conception and design, the analysis and interpretation of the data; drafting of the manuscript and revising it critically for important intellectual content. All authors have read and approved the final manuscript.

## Pre-publication history

The pre-publication history for this paper can be accessed here:


